# Supplementary material for: Cytoplasmic condensation induced by membrane damage is associated with antibiotic lethality
Source: Nat Commun. 2021 Apr 19;12:2321. doi: 10.1038/s41467-021-22485-6 (PMC8055701; doi:10.1038/s41467-021-22485-6)
Supplement: Supplementary file 1 — Supplementary Information [file 41467_2021_22485_MOESM1_ESM.pdf]

Supplementary Information: Cytoplasmic condensation induced by membrane damage is associated with antibiotic lethality

Felix Wong[1,2], Jonathan M. Stokes[1,2], Bernardo Cervantes[1,2,3], Sider Penkov[4], Jens Friedrichs[5], Lars D. Renner[5,*], and James J. Collins[1,2,6,*]

[1]Institute for Medical Engineering & Science and Department of Biological Engineering, Massachusetts Institute of Technology, Cambridge, MA 02139, USA
[2]Infectious Disease and Microbiome Program, Broad Institute of MIT and Harvard, Cambridge, MA 02142, USA
[3]Microbiology Graduate Program, Massachusetts Institute of Technology, Cambridge, MA 02139, USA
[4]Institute for Clinical Chemistry and Laboratory Medicine at the University Clinic and Medical Faculty of TU Dresden, 01307 Dresden, Germany
[5]Leibniz Institute of Polymer Research and the Max Bergmann Center of Biomaterials, 01069 Dresden, Germany
[6]Wyss Institute for Biologically Inspired Engineering, Harvard University, Boston, MA 02115, USA
[*]Corresponding authors: James J. Collins (jimjc@mit.edu) and Lars D. Renner (renner@ipfdd.de)

Supplementary Note 1

1.1 Generality of observed phenotypes

Here, we discuss the generality of results presented in the main text. In brief, we find evidence for the accumulation of reactive metabolic byproducts, but did not observe cytoplasmic condensation, in cells treated by β-lactams (Supplementary Fig. 1). Furthermore, we observed cytoplasmic condensation across different aminoglycosides and quinolones and in a different, Gram-positive species, *B. subtilis* (Supplementary Fig. 3).

1.1.1 β-lactam antibiotics

In *E. coli* cells treated by ampicillin, we observed increased fluorescence of carboxy-H_2DCFDA, DAF-FM, and C11-BODIPY relative to untreated controls (Supplementary Fig. 1c-e). However, we did not find significant cytoplasmic condensation in cells treated with either ampicillin or less lytic antibiotics such as mecillinam (Supplementary Fig. 1a,b), suggesting that typical cells likely undergo a different cell death pathway involving membrane bulging, as previous work has shown [1]. In contrast to kanamycin and ciprofloxacin, we found that ampicillin killing was less susceptible to glutathione protection (Supplementary Fig. 1f). A previous study [2] has shown that higher levels (50 mM) of glutathione pretreatment than those used in this work can better attenuate ampicillin killing, by as much as $\sim$3 logs; together with our results, these observations suggest glutathione protection in β-lactams to be largely concentration- and antibiotic-dependent.

1.1.2 Cytoplasmic condensation across different aminoglycoside and quinolone antibiotics, and also in *Bacillus subtilis*

In addition to kanamycin (Supplementary Fig. 3a) and ciprofloxacin (Fig. 1b of the main text), we observed similar cytoplasmic condensation in gentamicin- (an aminoglycoside) and norfloxacin- (a quinolone) treated *E. coli* (Supplementary Fig. 3b). The evolutionarily distant, Gram-positive rod *B. subtilis* also exhibited similar condensation and lysis and increased levels of lipid peroxidation, as assayed by C11-BODIPY, when treated with kanamycin and ciprofloxacin (Supplementary Fig. 3c). Intriguingly, the incidence of cytoplasmic condensation in *B. subtilis* was decreased relative to *E. coli* under both kanamycin and ciprofloxacin treatment. Furthermore, we observed significant membrane bulging in *B. subtilis* cells treated by ciprofloxacin (Supplementary Fig. 3c), reminiscent of bulging *E. coli* cells treated by β-lactams [1, 3]. The occurrence of membrane bulging in *B. subtilis* is surprising, since its turgor pressure is estimated to be $\sim$10-fold larger than that of *E. coli* [4], while the yield areal strains of bacterial membranes are anticipated to be similar [1, 5]. Thus, if the mechanical stresses in a membrane bulge were $pR/2$, where p is the turgor pressure and R is the radius of the bulge [1], then we would anticipate *B. subtilis* cells to lyse immediately after bulge formation due to membrane yielding. The occurrence of bulging in *B. subtilis* therefore suggests that, like *E. coli* (below), *B. subtilis* cells may also lose a significant amount of cellular turgor due to membrane damage as a result of ciprofloxacin treatment.

1.1.3 Relation to previous work

As a historical remark, we note that aspects of cytoplasmic condensation have been observed in previous work revealing the leakage of [^{3}H]-uracil-labeled cellular contents [6, 7] and formation of vacuoles [8] in quinolone-treated *E. coli*. However, to our knowledge, a physiological characterization of the condensed state, in addition to the relationship between condensation and turgor loss, cell death, reactive metabolic byproducts, and glutathione protection, has not, until now, been studied.

1.2 Biophysical model of cytoplasmic condensation

To better understand the physiology of the condensed state, we developed a biophysical model of cell envelope mechanics which predicts (1) smaller turgor and cytoplasmic condensation to arise from elastic relaxation to an equilibrium state, which is governed by flow of solutes outside the cell and induced by nanometer-scale membrane defects; and (2) the number of such defects consistent with the empirically

observed timescale and magnitude of condensation. Building on previous work [1], we model the Gram-negative bacterial cell envelope as the combination of an elastic shell (the cell wall) sandwiched between two fluid membranes (the inner and outer membranes). The free energy of the cell envelope and its enclosed volume includes both the elastic strain energies of all three layers and the entropy of mixing, primarily of solutes contained within:

$$\mathcal{F} = E^{w}_{\text{strain}} + E^{i}_{\text{strain}} + E^{o}_{\text{strain}} - TS. \tag{S1}$$

Here, the superscripts ^{w}, ^{i}, and ^{o} denote cell wall, inner membrane, and outer membrane quantities, respectively, E_{strain} is the elastic strain energy, T is the temperature, and S is the entropy of mixing water and solute molecules. For simplicity, we assume that there are only water molecules outside the cell; S can then be expressed as $S = -k(n_s \ln x_s + n_w \ln x_w)$, where k is Boltzmann's constant, x_s and x_w are the number fractions of solute and water molecules, respectively, and n_s and n_w are the numbers of solute and water molecules, respectively. We point out the following details regarding the model:

- The turgor pressure is defined as $p = kTC$, where C is the solute concentration. Note that the origin of turgor pressure is entropic, and it will therefore decrease continuously with flow of solutes outside the cell and flow of water into the cell (below).
- Throughout, we will neglect the hemispherical poles of the cell for simplicity and consider only the cylindrical bulk.
- We assume that the contributions of any disjoint, but potentially load-bearing cytoskeletal elements, such as MreB [9], are coarse-grained by the continuum description of the cell envelope layers above; the elastic moduli should therefore be viewed as effective ones.
- For simplicity, we will focus on modeling the cellular periplasm as, effectively, a rigid, permeable, and gel-like body that supports the inner membrane [10, 11, 12], but is not isosmotic with the cytoplasm [13]. A previous study combining diffusion measurements in the cytoplasm and periplasm and hyperosmotic shocks suggested that the periplasm is iso-osmotic with the cytoplasm, with the main turgor pressure drop occurring across the cell wall and outer membrane. However, as indicated by a subsequent study [11], in this case the bending energy of the inner membrane would be minimal for rod-like cells when the inner membrane assumes the shape of a cylinder with the largest radius possible. Equilibration of the inner membrane would therefore result in the inner membrane squeezing out the periplasm. A model in which the periplasm and cytoplasm are isosmotic, with no force exerted on the inner membrane by the periplasm, is therefore inconsistent with the existence of a periplasm. The periplasm may exist upon the inclusion of a membrane pinning potential [11, 12]; however, as noted before [11], for previously considered parameter values [12], the membrane pinning potential is large enough so that the periplasm is effectively a rigid body.
- Additionally, we assume that the periplasm does not hinder the transport of solutes and water out of the cytoplasm and can be viewed independently of each cell envelope component. We therefore neglect the periplasm in what follows.
- Finally, we note that, although molecules such as Braun's lipoprotein anchor the outer membrane to the cell wall, the estimated number of such outer membrane-wall anchors ($\sim 10^6$) are few in comparison to the estimated numbers ($\sim 10^7$) of phospholipids [1, 14, 15]. Hence, free phospholipids could modulate the reference membrane states and allow for membrane reorganization. That the cell envelope layers can slide with respect to each other will be relevant to the calculations in §1.2.2 below.

1.2.1 Change in cellular turgor and volume due to membrane defects

We hypothesize that membrane damage is well described by the appearance of nanoscale gaps in the membrane (Fig. 2a of the main text). Accordingly, we model membrane defects as holes with characteristic radius $r_d \approx 1$ nm, which is much smaller than the combined thickness of the inner and outer membranes, $\sim$20 nm. Assuming it to be laminar, the hydrodynamic flow of cytoplasmic contents from inside to outside

the cell is well described by Poiseuille flow, with a volumetric flow rate of

$$Q = \frac{\Delta P A^2}{8\pi\mu L_d}. \tag{S2}$$

Here ΔP is the pressure drop inside and outside the cell, $A = \pi r_d^2$ is the defect area, L_d is the defect length, and μ is the viscosity of the medium. Note that, due to the entropic origin of turgor, p decreases with flow of solutes outside the cell and flow of water into the cell through the semi-permeable cell membranes. In turn, the membrane defect radius will decrease with the turgor due to there being less mechanical strain in the cell membranes. Nonetheless, for characteristic parameter values, as summarized in Supplementary Table 2, we note that, at the start of flow, $Q \approx 10^{-21}$ m^3/s and the Reynolds number is $\mathrm{Re} = 2Qr_d\rho/(\mu A) \approx 10^{-6}$, where ρ is the density of water. This suggests, self-consistently, the flow to indeed be laminar. Furthermore, assuming this flow rate to be constant in time, a simple but surprising estimate shows that only ten such pores, corresponding to removal of only $\sim 0.0001\%$ of all phospholipids in a membrane layer, are sufficient to predict a flow comparable to the entire cellular volume out of the cell within one minute.

We now undertake a more detailed analysis, taking into account the decrease of turgor and defect radius with flow of solutes outside the cell. A characteristic value of the diffusion constant of ions in water is $D \approx 10^{-9}$ m^2/s [16], so that a typical root-mean-square distance traveled by an ion per second is 10 μm. Accordingly, we assume solutes to be significantly diluted once outside the cell, so that $\Delta P = p$, the turgor pressure of the cell. Viewing n_s, p, r_d, Q, and the cell volume, V, as time-dependent quantities that change with flow of solutes out of the cell, we therefore write:

$$Q(t) = \frac{\pi p(t) r_d(t)^4}{8\mu L_d}, \quad p(t) = \frac{kTn_s(t)}{V(t)}, \quad \frac{dn_s(t)}{dt} = -\frac{\mathcal{N}Q(t)n_s(t)}{V(t)}, \tag{S3}$$

where $\mathcal{N}$ is the number of such membrane defects. It remains to determine $V(t)$ and $r_d(t)$; this becomes a problem of elasticity.

1.2.2 Elastic determination of the cellular volume

Due to the possibility of water flow into the membrane as the number of solutes are modulated, we hypothesize that the cellular volume is determined by the equilibration of the elastic strain energies in Eq. (S1). In particular, given the turgor pressure, $p(t)$, the cell envelope is free to change its dimensions to minimize the free energy. The bulk flow of water through the cell membranes is described by

$$\frac{dV_{\mathrm{water}}}{dt} = L_p A_{\mathrm{cell}} p, \tag{S4}$$

where L_p is the hydraulic conductivity of the membranes and A_{cell} is the total membrane surface area [17]. For characteristic values of these parameters, as summarized in Supplementary Table 2, we find that a typical ∼40% change in cellular volume occurs within ∼1 s. Hence, for the timescale of interest (∼1 s) here, we find that water flow indeed occurs fast enough for the cell to be in equilibrium.

We therefore determine $V(t)$ and $r_d(t)$ by finding the elastic stresses in the equilibrium conformation. For this, we resort to a linear theory and assume a linear-elastic cell wall, with reference radius and lengths r_0^w and L_0^w, respectively, and (two-dimensional) Young's modulus and Poisson's ratio Y^w and ν^w, respectively. Moreover, we view the two membranes as materially identical and fluid in-plane, so that their stretching is governed by their area-stretch modulus, $K = K^i = K^o$, and excess reference surface area ratio, $\gamma = A_0^i/A_0^w - 1 = A_0^o/A_0^w - 1$, where A_0^w is the reference cell wall surface area, and A_0^i and A_0^o are the inner and outer membrane reference surface areas, respectively [1]. Ignoring bending terms, which are anticipated to be dominated by the stretching terms [1], the free energy of Eq. (S1) can then be expressed as

$$\mathcal{F} = \frac{1}{2Y^w}\int [(\sigma_{xx}^w)^2 + (\sigma_{yy}^w)^2 - 2\nu^w \sigma_{xx}^w \sigma_{yy}^w] dA^w + 2K\int (u^i)^2 dA^i + 2K\int (u^o)^2 dA^o - TS, \tag{S5}$$

where the integrals are over the deformed surface areas, σ^w_{xx} and σ^w_{yy} are cell wall stresses, and u^i and u^o are inner and outer membrane strains, respectively. Note that the form of E^i_{strain} and E^o_{strain} in Eq. (S5) arises from the fluid in-plane nature of the membranes; it follows from this that the membrane strains and stresses are isotropic and spatially homogeneous [1]. As the cell wall is cylindrical, its strains and stresses will also be spatially homogeneous, but not necessarily isotropic.

Depending on the values of γ, K, and p, we note that the deformed membrane dimensions may be different from each other and those of the cell wall: in the limit of small $-1 < \gamma \ll 0$ and $p\ell/K \ll 1$ for a characteristic membrane length ℓ, for instance, the free energy is minimal when the inner membrane forms a spherical vesicle inside the cell and the cell wall and outer membrane bear no load. However, we may anticipate a parameter regime in which each envelope layer bears some load (below). Then, by symmetry of the inner and outer membranes, $u = u^i = u^o$ and the membrane stresses $\sigma = \sigma^i = \sigma^o$; moreover, these quantities will all be nonzero. In general, the mechanical stresses will be related to the strains by the following constitutive relations [1]:

$$\sigma^w_{xx} = \frac{Y^w}{1-(\nu^w)^2}(u^w_{xx} + \nu^w u^w_{yy}), \quad \sigma^w_{yy} = \frac{Y^w}{1-(\nu^w)^2}(u^w_{yy} + \nu^w u^w_{xx}), \quad \sigma = 2Ku. \tag{S6}$$

Here, the cell wall strains u^w_{xx} and u^w_{yy} correspond to the stresses σ^w_{xx} and σ^w_{yy}. Furthermore, the linear strain-displacement relations are

$$u^w_{xx} = \frac{r - r^w_0}{r^w_0}, \quad u^w_{yy} = \frac{L - L^w_0}{L^w_0}, \quad u = \frac{A^i - A^i_0}{2A^i_0} = \frac{A^o - A^o_0}{2A^o_0}, \tag{S7}$$

where r and L are the deformed cell wall radius and length, respectively. Assuming that the membranes share the same deformed radius and length (below), we substitute Eqs. (S6) and (S7), as well as the relation $n_w = \pi r^2 L/m_w$, where m_w is the volume occupied per water molecule, into Eq. (S5). From this, we find that $\mathcal{F}$ can be rewritten as a function of two unknowns, r and L, and several parameters including the elastic constants, γ, and n_s. Hence, we will minimize $\mathcal{F}$ over r and L, from we determine all associated elastic quantities.

As mentioned above, we anticipate that, for typical cells, the membrane reference areas will be similar to that of the cell wall, so that $|\gamma| \ll 1$ [1]. Furthermore, we anticipate all cell envelope layers to be load-bearing and in contact in the deformed state, so that we may suppose a common value of the deformed cell length and radius among all envelope layers; these may be expressed as $L = L^w_0 + \delta L$ and $r = r^w_0 + \delta r$, where δL and δr are viewed as small relative to L^w_0 and r^w_0. Next, we make the following small-variable assumptions: $n_s/n_w \ll 1$ and $\delta r/r, \delta L/L = O(\varepsilon)$, where $\varepsilon \ll 1$, consistent with the linear theory. In particular, we will expand $\mathcal{F}$ to first order in γ, first order in n_s, and second order in ε. Doing so, and analytically solving for the values of δL and δr which minimize $\mathcal{F}$, upon substitution of the solution into Eqs. (S6) and (S7) we find

$$\begin{aligned}\sigma^w_{xx} &= \frac{\gamma K Y^w}{Y^w + 2K(1-\nu^w)} + \frac{kTn_s[K(1-\nu^w) + 2Y^w]}{2\pi r^w_0 L^w_0[2K(1-\nu^w) + Y^w]} \\ &\quad + O(\varepsilon^2) + O\left[\left(\frac{n_s}{n_w}\right)^2\right] + O(\gamma^2) + O(\gamma\varepsilon) + O\left(\frac{\gamma n_s}{n_w}\right) + O\left(\frac{\varepsilon n_s}{n_w}\right).\end{aligned} \tag{S8}$$

Accurate to the same order, we have

$$\sigma^w_{yy} = \frac{\gamma K Y^w}{Y^w + 2K(1-\nu^w)} + \frac{kTn_s[K(1-(\nu^w)^2) + Y^w]}{2\pi r^w_0 L^w_0[2K(1-\nu^w) + Y^w]}, \quad \sigma = \frac{K[3kTn_s(1-\nu^w) - 2\pi r^w_0 L^w_0 \gamma Y^w]}{4\pi r^w_0 L^w_0[2K(1-\nu^w) + Y^w]}. \tag{S9}$$

It is straightforward to verify that $\sigma^w_{xx} + 2\sigma = \frac{kTn_s}{\pi r_0 L_0}$ and $\sigma^w_{yy} + 2\sigma = \frac{kTn_s}{2\pi r_0 L_0}$, so that Laplace's law [1] is satisfied. Furthermore, when $K = 0$, the membrane stress $\sigma = 0$ and we recover the cylinder stresses $\sigma^w_{xx} = \frac{kTn_s}{\pi r_0 L_0}$ and $\sigma^w_{yy} = \frac{kTn_s}{2\pi r_0 L_0}$ in the cell wall.

Finally, by viewing the stresses in Eqs. (S8) and (S9) as functions of time through their dependence on $n_s = n_s(t)$ and finding the corresponding time-dependent strains through the linear constitutive relations of Eq. (S6), we can write closed-form expressions for the following:

$$V(t) = \pi(r_0^w)^2 L_0^w [1 + 2u_{xx}^w(t) + u_{yy}^w(t)], \quad r_d(t) = r_d^0(1 + u(t)). \tag{S10}$$

Here r_d^0 denotes the reference (unstretched) radius of the membrane defect, and henceforth all equalities will be accurate to the orders shown in Eq. (S8).

1.2.3 The final dynamical equation

Iteratively substituting Eqs. (S6)-(S10) into Eq. (S3), we find that a single equation governs the dynamics of solute flow which, in turn, determines all other quantities:

$$\frac{dn_s(t)}{dt} = -\frac{\mathcal{N}kTn_s(t)^2(Y^w)^2[2\pi r_0^w L_0^w r_d^0(8K(1-\nu^w) + (4-\gamma)Y^w) + 3kTr_d^0(1-\nu^w)n_s(t)]^4}{\Phi(t)}, \tag{S11}$$

where $\Phi(t) = 8192\pi^3\mu L_d(r_0^w)^6(L_0^w)^4[2K(1-\nu^w)+Y^w]^2[2\pi r_0^w L_0^w Y^w(K(2+3\gamma)(1-\nu^w)+Y^w)+kTn_s(t)(K(1-(\nu^w)^2)+(5-4\nu^w)Y^w)]^2$, and $n_s(t=0) = n_s^0$, the initial number of solutes inside the cell. This is a complicated ordinary differential equation involving a degree-six rational function that is difficult to solve analytically or approximately for the parameter values of interest. We therefore turn to numerical solutions of this equation for these parameter values (Supplementary Table 2).

1.2.4 The equilibrium state: timescales of equilibration and cellular morphology

Solving the dynamical Eq. (S11) numerically for the parameter values summarized in Supplementary Table 2, we find that the model predicts a cellular volume shrinkage of ~20% over the empirically observed, minute-timescale of condensation, consistent with experimental observations (Fig. 2b-f of the main text). For this to occur, the model shows that the turgor pressure is essentially abolished on the minute timescale (Fig. 2e of the main text), consistent with our osmotic shock experiments (Fig. 1e of the main text and Supplementary Fig. 5). Furthermore, the model predicts that the condensed state is not only a state of mechanical equilibrium, but also a steady state ($t \to \infty$) maintained by increasingly small solute leakage and cellular volume shrinkage (Fig. 2d,f of the main text). Indeed, the model predicts that there remains cytoplasmic material in condensed cells. This is consistent with fluorescence microscopy (Fig. 1b of the main text) and AFM measurements, which show the elastic moduli of a typical condensed cytoplasm to be larger than that of the shrunken region (Fig. 1f of the main text and Supplementary Fig. 7). We note here that these model predictions are general across a range of different membrane defect sizes and, for different defect sizes, predict different numbers of membrane defects to form (Supplementary Fig. 10).

For $\gamma < 0$ and vanishingly small turgor pressures corresponding to large t (Fig. 2e of the main text), we note that the inner membrane may form invaginations and retract from the cell wall, consistent with Fig. 1b of the main text. In this case, the assumption that all envelope layers are load-bearing no longer holds. The accuracy of the model at long times therefore depends on γ; nonetheless, for $\gamma \approx 0$, as may be expected for *E. coli* [1], we expect the model to be accurate and robust at the onset of condensation, at which point all envelope layers are in contact and load-bearing.

Finally, we note that, in Eq. (S5) the mixing of solutes and water inside the cell is assumed to be homogeneous, and the free energy depends on the cell wall stresses, the cell wall area, the cell membrane areas, and the cellular volume. Consistent with ignoring bending terms in this expression, it is possible that the cell membranes may assume different shapes, provided that the remaining quantities are conserved. Accordingly, the model does not discriminate between condensed phenotypes wherein the phase-light region appears mid-cell or at the poles, and whether phase-dark regions of condensed cells may be connected. We anticipate future experimental studies to determine the factors that determine where, along a cell, condensation is initiated.

Supplementary Tables

**Antibiotic**	**Empirical MIC Range (μg/mL)**	**Working MIC (μg/mL)**
Kanamycin	*E. coli* MG1655: [5.0,12.0]	5.0
	E. coli BW25113: [5.0,6.25]	
Ciprofloxacin	*E. coli* MG1655: [0.03125,0.125]	0.1
	E. coli BW25113: [0.03125,0.125]	
	E. coli Δ*gshA*: [0.03125,0.125]	
	E. coli Δ*gor*: [0.03125,0.125]	
Gentamicin	*E. coli* MG1655: [3.13,6.25]	5.0
	E. coli BW25113: [3.13,6.25]	
	E. coli Δ*gshA*: [3.13,6.25]	
	E. coli Δ*gor*: [3.13,12.5]	
Norfloxacin	*E. coli* MG1655: [0.15,0.5]	0.5
Ampicillin	*E. coli* MG1655: [3.13,12.5]	10.0
	E. coli BW25113: [3.13,12.5]	
	E. coli Δ*gshA*: [3.13,12.5]	
	E. coli Δ*gor*: [3.13,12.5]	
Mecillinam	*E. coli* MG1655: [0.63,2.0]	1.0
**With 10 mM glutathione**		
Kanamycin	*E. coli* MG1655: [39.0,78.0]	40.0
Ciprofloxacin	*E. coli* MG1655: [0.156,0.3125]	0.3
**With 10 mM dithiothreitol**		
Kanamycin	*E. coli* MG1655: [5.0,12.5]	5.0
Ciprofloxacin	*E. coli* MG1655: [0.03125,0.1]	0.1
**With 10 mM mercaptoethanol**		
Kanamycin	*E. coli* MG1655: [5.0,12.5]	5.0
Ciprofloxacin	*E. coli* MG1655: [0.03125,0.1]	0.1
**With 50 mM α-tocopherol**		
Kanamycin	*E. coli* MG1655: [5.0,12.5]	5.0
Ciprofloxacin	*E. coli* MG1655: [0.03125,0.1]	0.1

Supplementary Table 1: **Minimal inhibitory concentrations (MICs) of antibiotics used in this study.** Ranges were determined from three replicates for each culture dilution (1:100 and 1:10,000) and each growth vessel (14-mL Falcon tubes and 96-well plates); see *Methods* for details. Identical working MICs were used across all strains due to the similarity in observed empirical MIC ranges. Similar empirical MICs were observed, and the same working MICs for *E. coli* MG1655 used, for *B. subtilis* 168.

Variable	Value	Reference
Viscosity of water, μ	8.9×10^{-4} Pa $\cdot$ s	–
Density of water, ρ	997 kg/m^3	–
Volume occupied per water molecule, m_w	3×10^{-29} m^3	–
Temperature, T	300 K	This work
Characteristic (reference) membrane defect radius, r_d^0	1 nm	This work
Characteristic membrane defect length, L_d	20 nm	This work
Characteristic number of membrane defects, $\mathcal{N}$	10	This work
Membrane bilayer thickness	10 nm	[18]
Membrane hydraulic conductivity, L_p	10^{-12} m^3/N $\cdot$ s	[17, 19]
Characteristic *E. coli* (reference) cell wall radius, r_0^w	0.5 μm	This work
Characteristic *E. coli* (reference) cell wall length, L_0^w	2 μm	This work
E. coli cell wall two-dimensional Young's modulus, Y^w	0.2 N/m	[1, 20, 21, 22]
E. coli cell wall Poisson's ratio, ν^w	0.2	[1, 22]
E. coli membrane area-stretch modulus, K	0.1 N/m	[1, 5]
E. coli membrane excess reference surface area ratio, γ	0	This work; estimate from [1]
E. coli (initial) turgor pressure, p	10^5 Pa	[1, 20, 23]
E. coli (initial) number of solutes, n_s^0	3.8×10^7	$p = kTn_s/[\pi(r_0^w)^2 L_0^w]$

Supplementary Table 2: **Biophysical model parameters.**

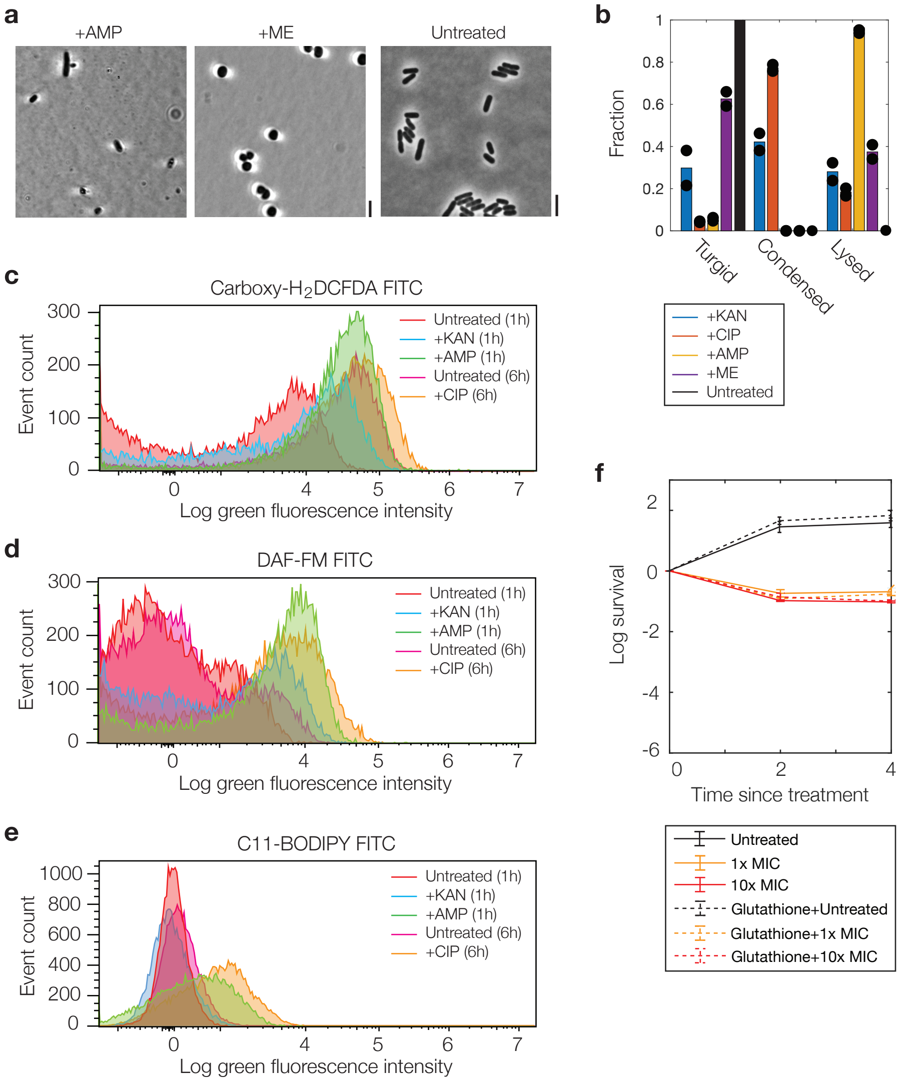

Supplementary Figure 1: **Generality of results to β-lactam antibiotics.**
a, Phase contrast microscopy images of *E. coli* cells treated by the β-lactam antibiotics ampicillin (AMP; left) and mecillinam (ME; right) 1 h after treatment, along with untreated control cells. Here and below, 10x MIC was used for all antibiotics, and scale bars indicate 3 μm.
b, Frequency of condensation and lysis observed under β-lactam treatment, compared to kanamycin, ciprofloxacin and an untreated control, after 6 h. Data are from two different fields of view from two biological replicates, and individual datapoints corresponding to each field of view are shown. Bars indicate averages. Number of cells in each field of view: kanamycin, 105 and 93; ciprofloxacin, 241 and 198; ampicillin, 21, 32, and 35; mecillinam, 91 and 98.
c-e, Histograms of fluorescence intensities of populations of control and antibiotic-treated cells, as assayed by flow cytometry using the fluorochromes indicated, in the presence of carboxy-H_2DCFDA, DAF-FM, and C11-BODIPY. Antibiotic-treated cells were treated for 1 h (ampicillin, kanamycin) or 6 h (ciprofloxacin); as in the main text, a longer treatment time was chosen for ciprofloxacin due to a majority of condensation events occurring later after treatment. Data representative of four biological replicates and 20,000 scattering events for each distribution.
f, Survival curves under ampicillin treatment with and without exogenous supplementation of 10 mM glutathione, as determined by CFU measurements, at 1x and 10x MIC. Control experiments are also shown. Each point represents two biological replicates, error bars indicate one standard deviation, and data are presented as mean values +/- SEM.

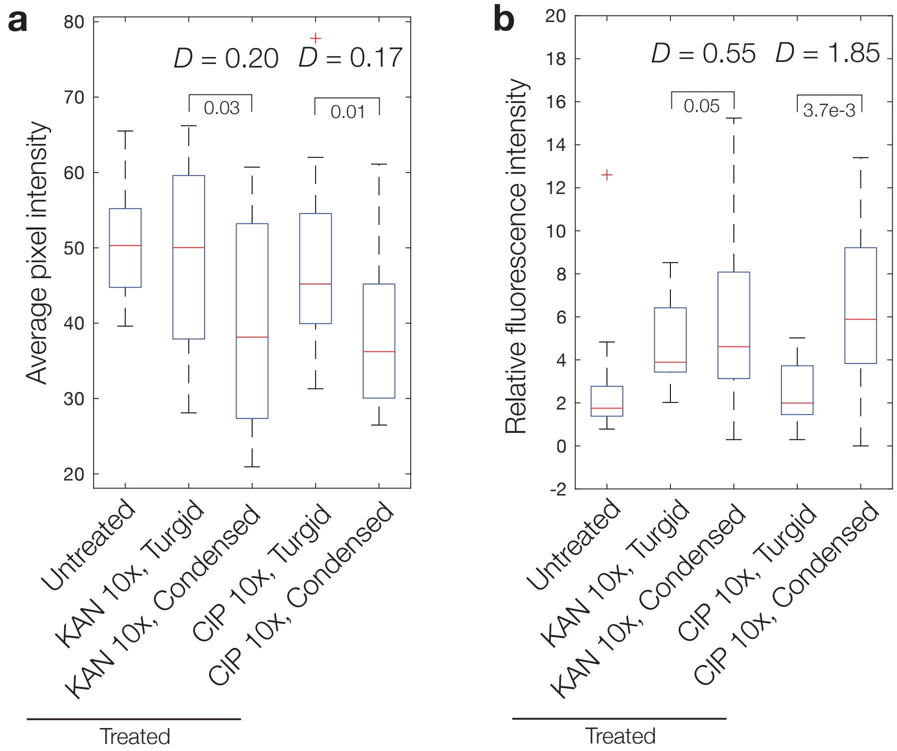

Supplementary Figure 2: **Phase contrast measurements of condensation indicate increased cytoplasmic density in condensed cells.**
a, Quantitative comparison of average pixel intensities before and after cytoplasmic condensation for *E. coli* cells treated by kanamycin (10x MIC) and ciprofloxacin (10x MIC), along with control cells. Data are representative of three different fields of view with 20 cells in each group. D denotes the population-average relative decrease in the average pixel intensity of a cell. Here and below, box plots indicating the median (center), 25th percentile and 75th percentile (bounds of box), and extreme data points not considered outliers (bounds of whiskers) are shown, and red crosses indicate outliers including the minimum and maximum values. p-values for one-sample t-tests for the decrease in the average pixel intensity are shown next to corresponding brackets.
b, Same as **a**, but for relative fluorescence intensities in the cytoplasmic mCherry strain.

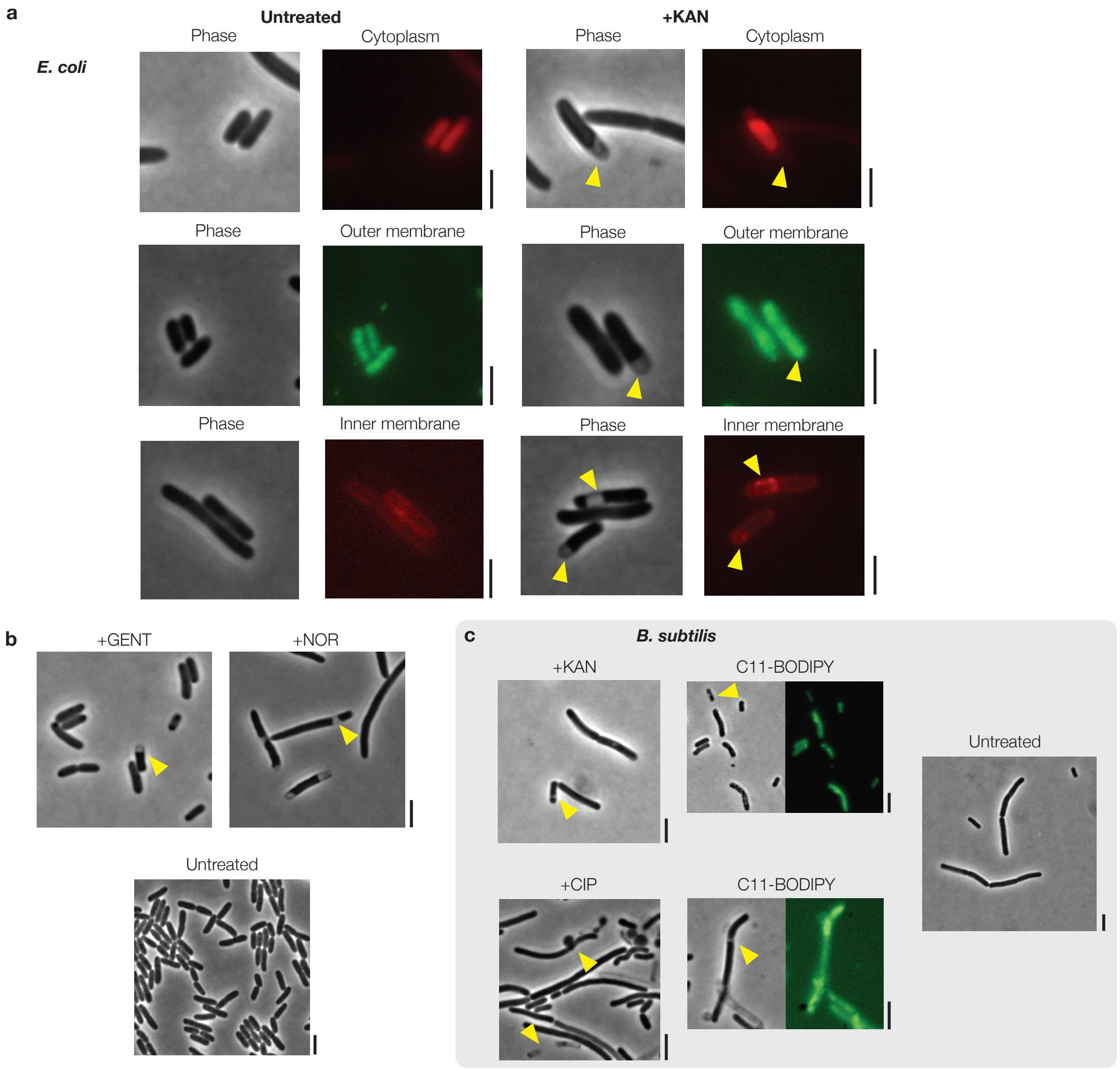

Supplementary Figure 3: **Generality of observed phenotypes to different antibiotics and bacterial species.**
a, *E. coli* cells with fluorescent cytoplasmic (mCherry), outer membrane (GFP), and inner membrane (mCherry) markers, as described in the *Methods*, with and without kanamycin treatment for 2 h at 10x MIC. Here and below, scale bars indicate 3 μm, imaging was performed in phase contrast and epifluorescence, and results are representative of two biological replicates. Yellow markers highlight condensed cells.
b, *E. coli* cells treated with other antibiotics for 2 h at 10x MIC: gentamicin (GENT), an aminoglycoside, and norfloxacin (NOR), a quinolone. Results are representative of two biological replicates, and yellow markers highlight condensed cells. A phase contrast microscopy image of corresponding untreated control cells is shown.
c, *B. subtilis* cells treated with kanamycin (50 μg/mL) and ciprofloxacin (1.0 μg/mL) after 2 h, with (right) and without (left) the dye C11-BODIPY. Results are representative of two biological replicates, and yellow markers highlight condensed, bulged, or lysed cells. A phase contrast microscopy image of corresponding untreated control cells is shown.

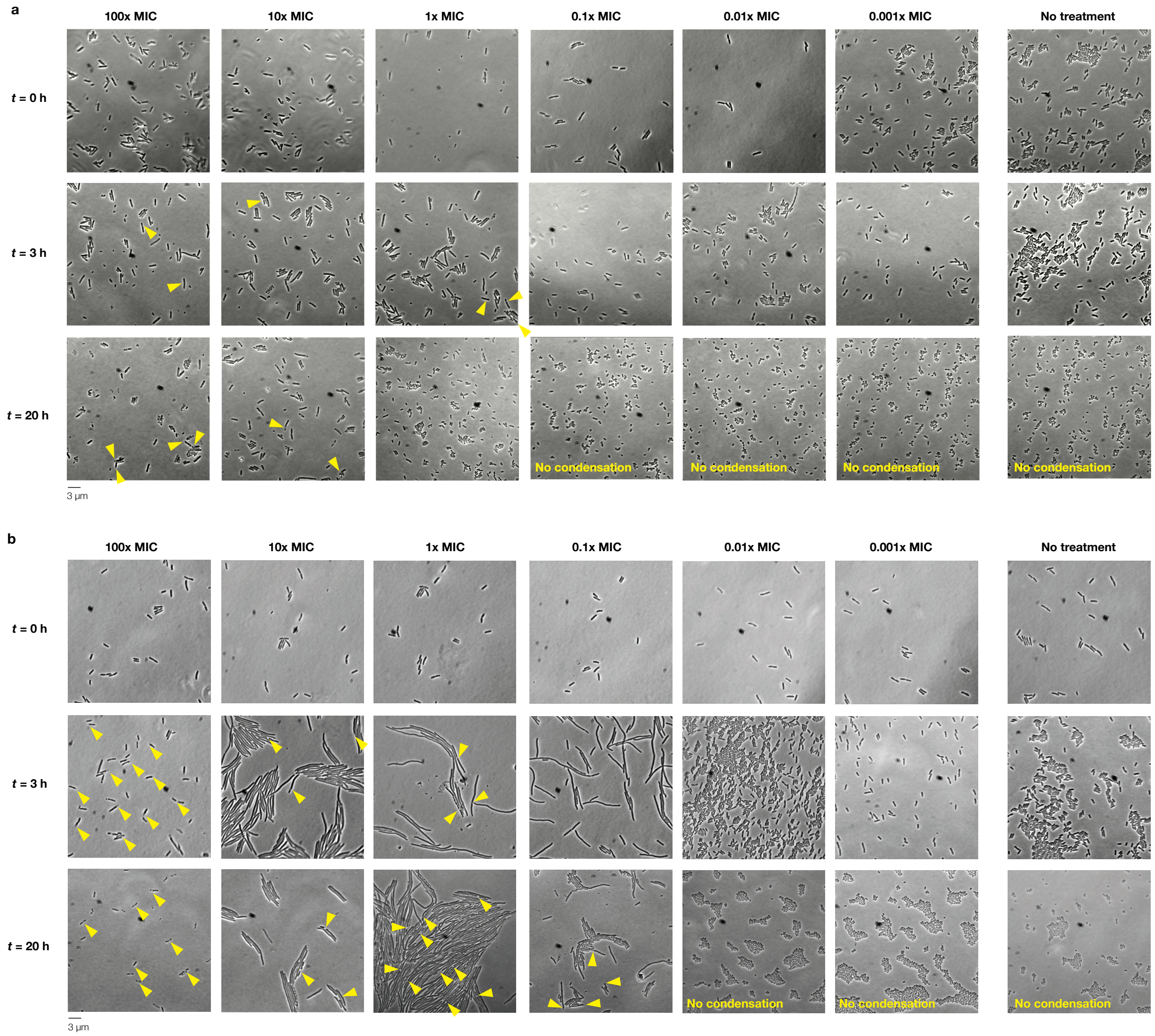

Supplementary Figure 4: **Generality of the condensation phenotype across different antibiotic concentrations.**

a, Phase contrast microscopy images of *E. coli* cells treated by various concentrations of kanamycin in culture as indicated, for various treatment times as indicated before imaging. Here time 0 refers to immediately before treatment. Each image is representative of three biological replicates, and yellow markers indicate a subset of condensed cells.

b, Same as panel **a**, but for ciprofloxacin.

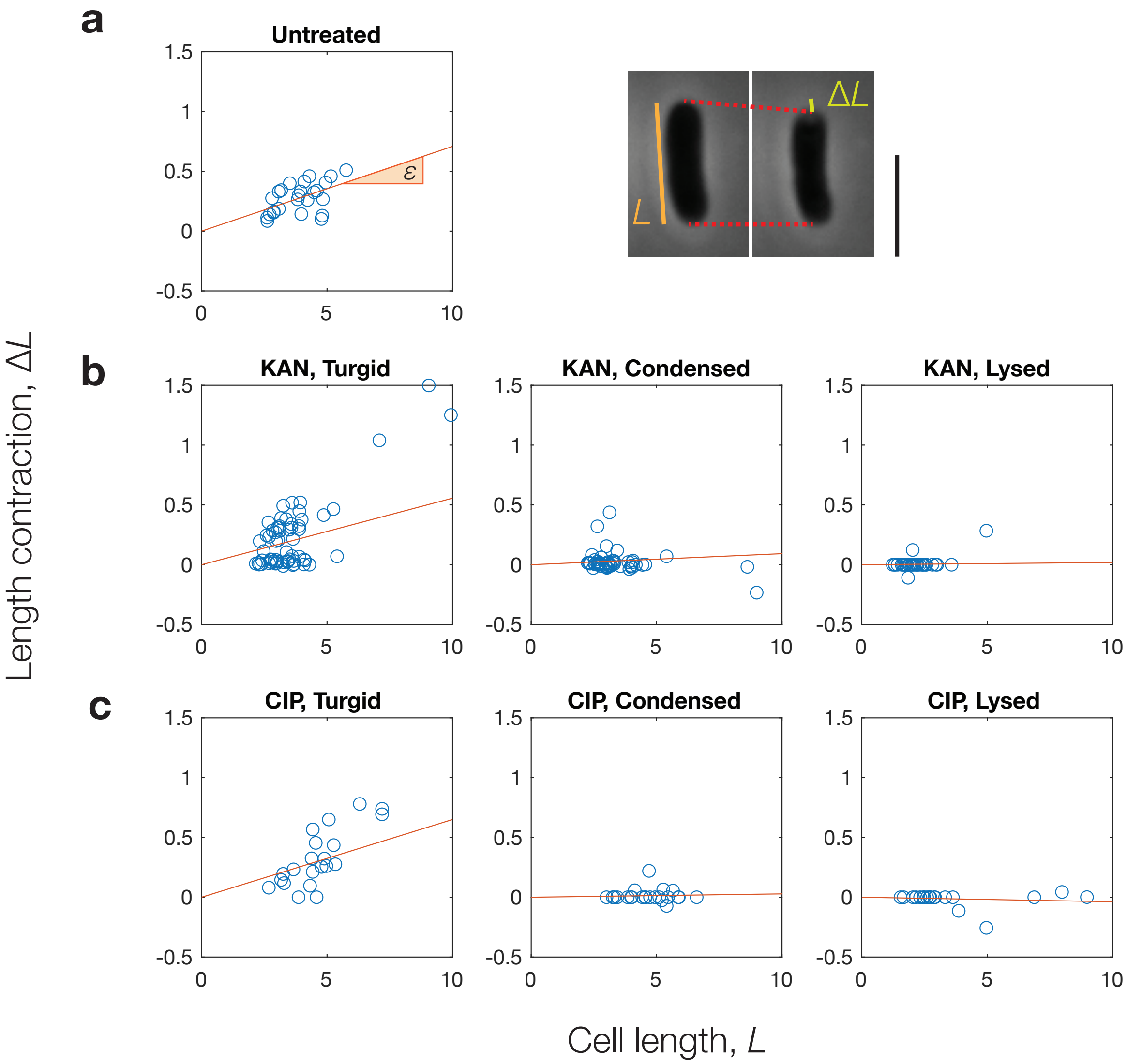

Supplementary Figure 5: **Osmotic shocks reveal collapse of cellular turgor.**
a, Plot of pre-shock cell length against post-shock length contraction for the osmotic shock experiments summarized in Fig. 1e of the main text, showing approximately linear relationships. Control cells with no antibiotic treatment were used. The average percentage length contraction, or strain, ε, is the slope of the best fit line. (Right) Representative phase contrast microscopy images show the response of an untreated cell. Scale bar, 3 μm.
b-c, Same as panel **a**, but for cells treated by kanamycin (10x MIC; **b**) or ciprofloxacin (10x MIC; **c**) and classified by phenotype.

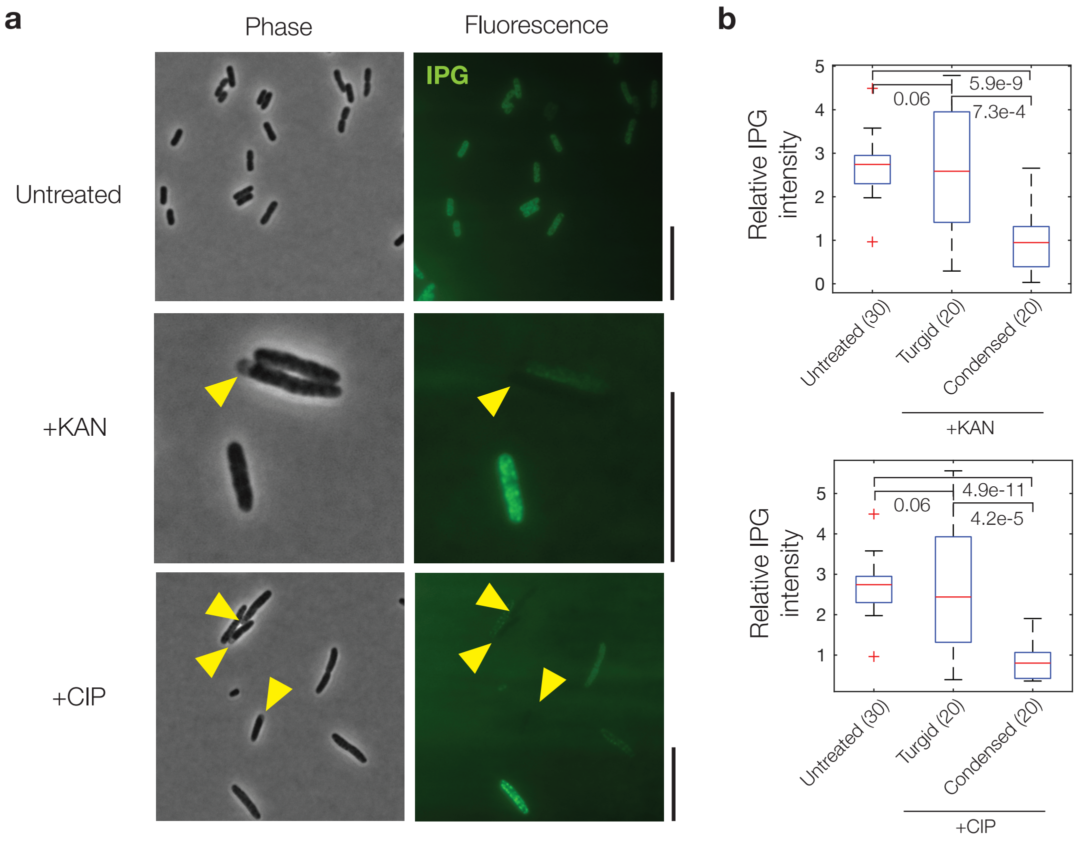

Supplementary Figure 6: **Measurements of IPG fluorescence.**
a, Phase-contrast and fluorescence microscopy images of control and antibiotic-treated cells (10x MIC) in the presence of IPG, a membrane-permeable potassium-sensitive dye. Cells were imaged at a time corresponding to 3 h of antibiotic treatment. Results are representative of two biological replicates, and yellow markers highlight condensed cells. Scale bars, 10 μm.
b, Fluorescence intensities of control and antibiotic-treated cells in the presence of IPG. Box plots indicating the median (center), 25th percentile and 75th percentile (bounds of box), and extreme data points not considered outliers (bounds of whiskers) are shown, and red crosses indicate outliers including the minimum and maximum values. The numbers of cells in each group are indicated in parentheses, and p-values for two-sample Kolmogorov-Smirnov tests are shown next to corresponding brackets.

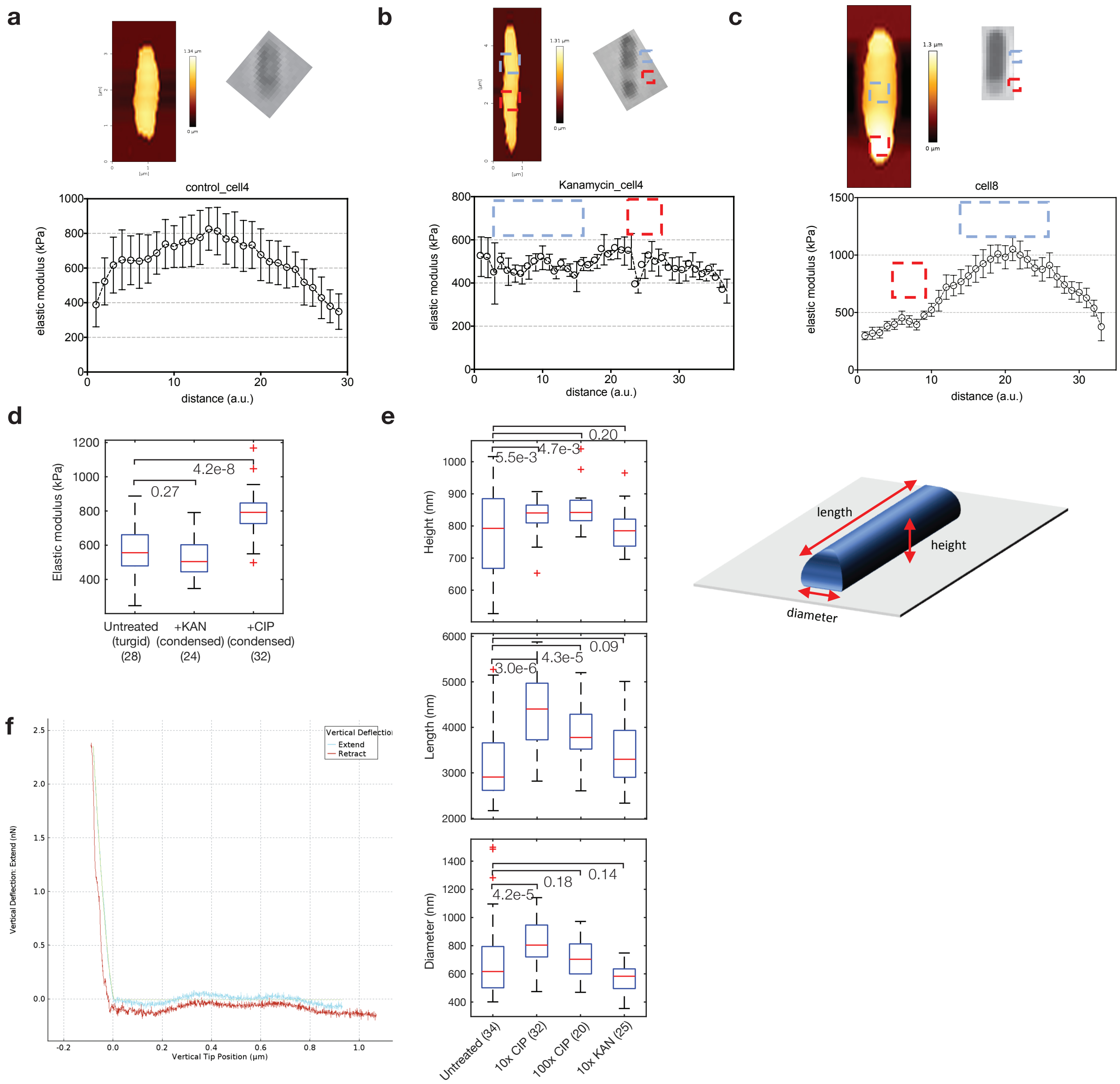

Supplementary Figure 7: **Details of the AFM experiments.**
a-c, Heatmaps of cell height (top) and plots of the elastic modulus (bottom) along the length of a cell (a.u.), for a representative untreated cell (**a**), a kanamycin-treated cell (10x MIC; **b**), and a ciprofloxacin-treated cell (10x MIC; **c**). Cells were assayed corresponding to a time ∼3 h of antibiotic treatment. Error bars indicate one standard deviation from at least two technical replicates for the same cell, and data are presented as mean values +/- SEM. Colored boxes highlight cellular regions.
d, Comparison of cell-averaged elastic moduli across different treatment conditions (10x MIC). Here and below, box plots indicating the median (center), 25th percentile and 75th percentile (bounds of box), and extreme data points not considered outliers (bounds of whiskers) are shown, and red crosses indicate outliers including the minimum and maximum values. The numbers of cells in each group are indicated in parentheses, and p-values for two-sample Kolmogorov-Smirnov tests are shown next to corresponding brackets.
e, (Left) Similar to **d**, but for inferred cellular dimensions across different treatment conditions (10x MIC). (Right) Schematic of the different cellular dimensions measured.
f, Sample force-distance curves from untreated control cells.

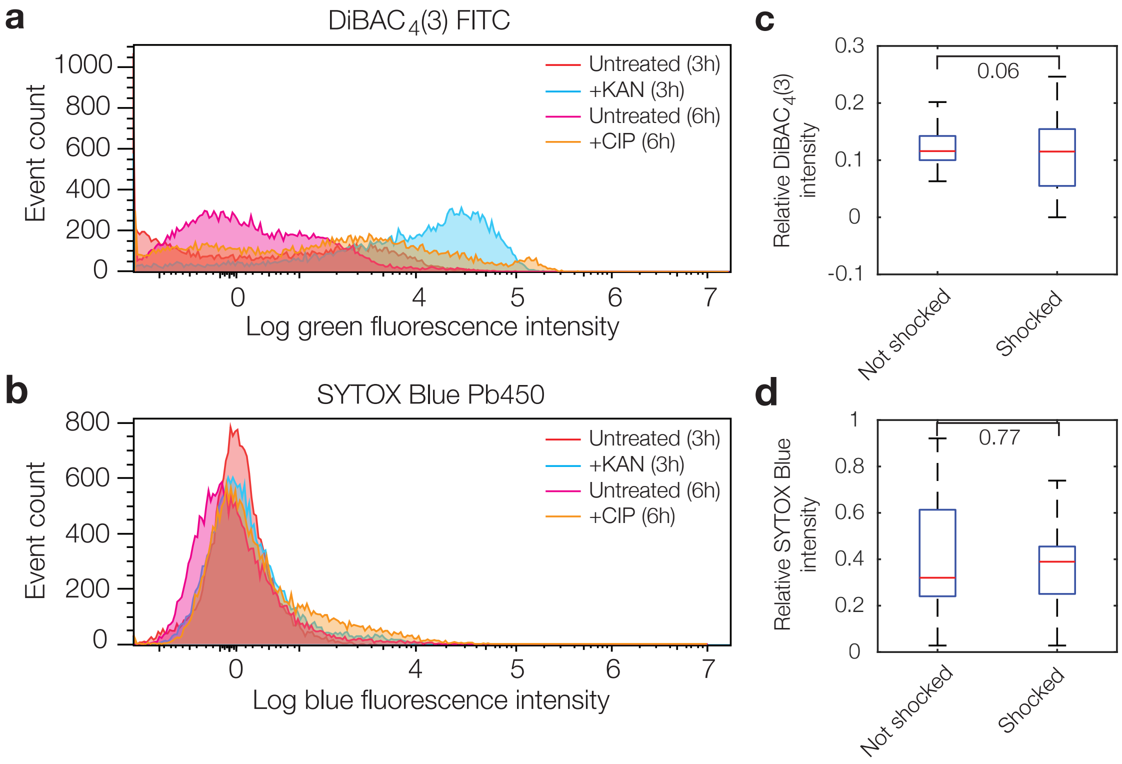

Supplementary Figure 8: **Additional DiBAC$_4$(3) and SYTOX Blue measurements.**
a-b, Histograms of fluorescence intensities of populations of control and antibiotic-treated cells in the presence of DiBAC$_4$(3) (**a**) and SYTOX Blue (**b**) 3 h after treatment (kanamycin) and 6 h after treatment (ciprofloxacin); as in the main text, a longer treatment time was chosen for ciprofloxacin due to a majority of condensation events occurring later after treatment. Concentrations used were 10x MIC for both antibiotics. Data representative of four biological replicates and 20,000 scattering events for each distribution. The fluorochromes used (FITC and Pb450) are indicated.
c-d, Fluorescence intensities of hyperosmotically shocked (500 mM sorbitol) cells in the presence of SYTOX Blue (**c**) and DiBAC$_4$(3) (**d**). Fluorescence images were taken immediately after shock. Data are from 20 cells in each group. Box plots indicating the median (center), 25th percentile and 75th percentile (bounds of box), and extreme data points not considered outliers (bounds of whiskers) are shown, and red crosses indicate outliers including the minimum and maximum values. p-values for two-sample Kolmogorov-Smirnov tests are shown next to corresponding brackets.

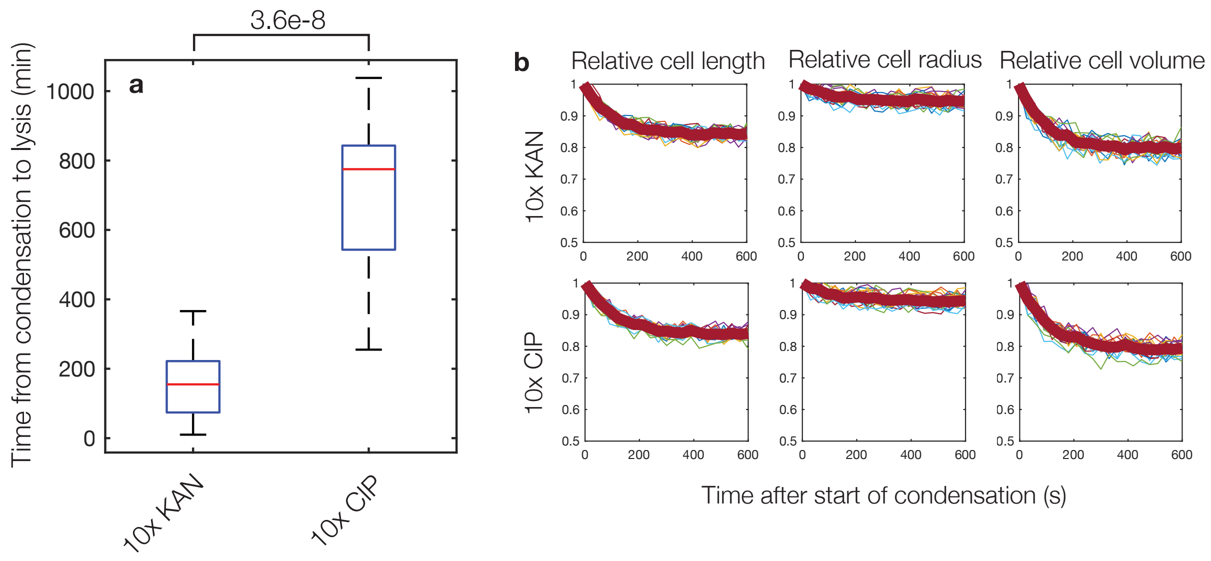

Supplementary Figure 9: **Times from condensation to lysis in antibiotic-treated cells and cellular dimensions of condensed cells.**

a, Times from condensation to lysis in kanamycin and ciprofloxacin-treated cells (10x MIC). Data are from 20 cells in each group. Box plots indicating the median (center), 25th percentile and 75th percentile (bounds of box), and extreme data points not considered outliers (bounds of whiskers) are shown, and red crosses indicate outliers including the minimum and maximum values. The p-value for a two-sample Kolmogorov-Smirnov test is shown next to the corresponding bracket.

b, Changes in relative cell length, cell radius, and cell volume across time, from the beginning of condensation events, in kanamycin and ciprofloxacin-treated cells (10x MIC), corresponding to Fig. 2f in the main text. Data are from 20 cells in each group (colored curves), and population-averaged traces (thick red curves) are shown. Note that fluctuations in individual traces arise due to variations in semi-automated image processing; see *Methods* for details of the image analysis.

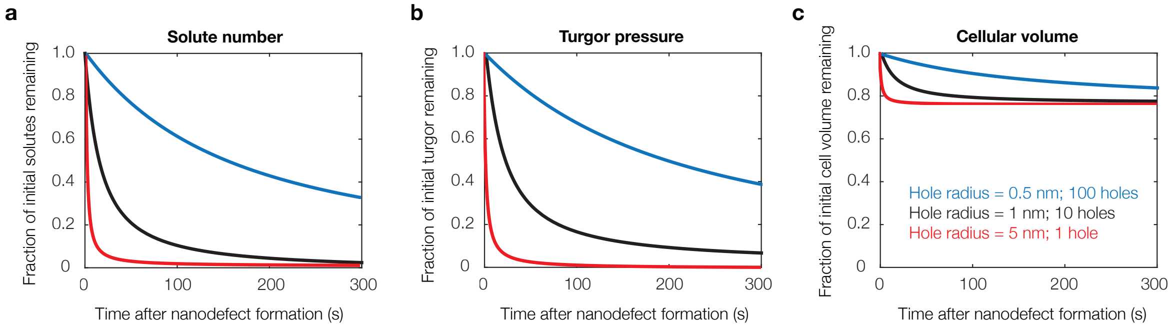

Supplementary Figure 10: **Sensitivity analysis of the model of solute outflow.**
a, Model predictions for the solute number as a function of time across different membrane defect radii (different colors). Depending on the size of typical membrane defect (0.5 nm, 1 nm, 5 nm radius), the model suggests that different numbers of membrane defects form (100, 10, and 1, respectively) as to be consistent with the minute-timescale of condensation. In all cases, the model predicts cells to condense after outflow of solutes from a cell, as detailed in Supplementary Note 1.
b-**c**, Same as panel **a**, but for the predicted cellular turgor pressure (**b**) and volume (**c**).

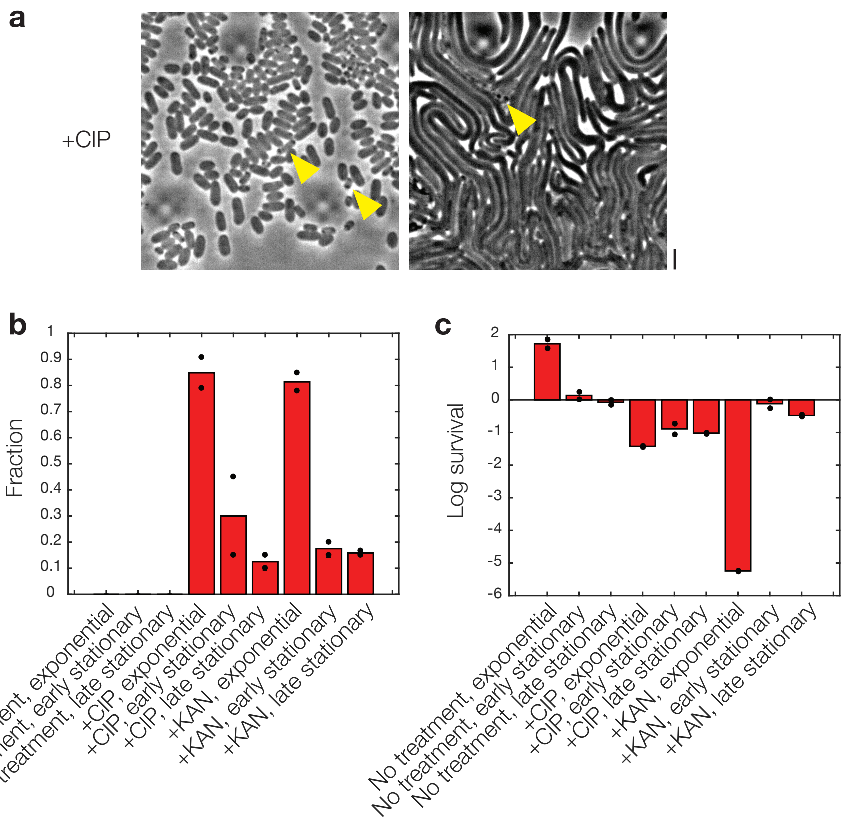

Supplementary Figure 11: **Experiments on stationary-phase cells.**
a, Microscopy images of early stationary-phase ($OD_{600} \approx 1.5$) *E. coli* cells treated by ciprofloxacin (10x MIC) at times 0 h (left) and 6 h (right). Results are representative of two biological replicates, and yellow markers indicate condensed or lysed cells. Scale bar, 3 μm.
b, Fractions of all cells that are condensed or lysed corresponding to the experiments in panel **a**, from two different fields of view of two biological replicates with at least 20 cells each, in addition to cells from early exponential phase ($OD_{600} \approx 0.1$) and late stationary phase ($OD_{600} > 2.0$), as described in the *Methods*. All antibiotics refer to 10x MIC, and "no treatment" cells were not treated by antibiotics. Individual points corresponding to each field of view are shown. The numbers of cells in each of two fields of view, according to the order of treatment shown along the horizontal axis, was as follows: 24 and 30, 56 and 76, 101 and 94, 195 and 206, 20 and 20, 20 and 20, 95 and 107, 20 and 20, 20 and 30.
c, Survival of *E. coli* cells treated by kanamycin and ciprofloxacin (10x MIC) 6 h after treatment, as determined by CFU counting, corresponding to panel **b**. Positive values of the log survival indicate an increase in CFU/mL. Results are from two biological replicates (individual points), and bars indicate averages.

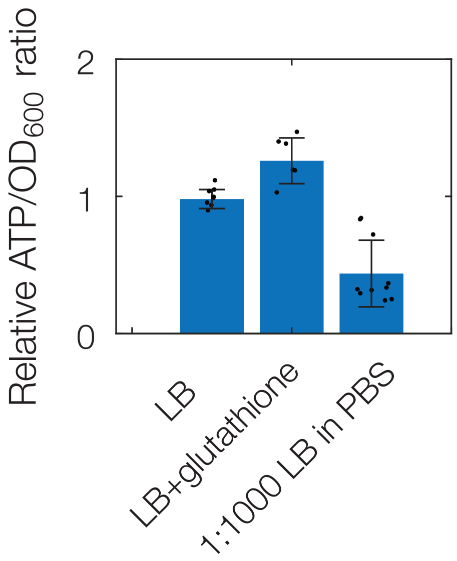

Supplementary Figure 12: **Bulk culture measurements of ATP abundance in cell cultures with and without glutathione pretreatment (10 mM).** Cells were assayed in early log phase ($OD_{600} \approx 0.1$). For comparison, measurements for cells grown in LB diluted 1:1000 in PBS are shown. Error bars indicate one standard deviation, bars indicate averages, and data are presented as mean values +/- SEM. Individual measurements from biological replicates are shown as black points. Number of biological replicates in each condition: LB, 8; glutathione, 6; 1:1000 LB in PBS, 10.

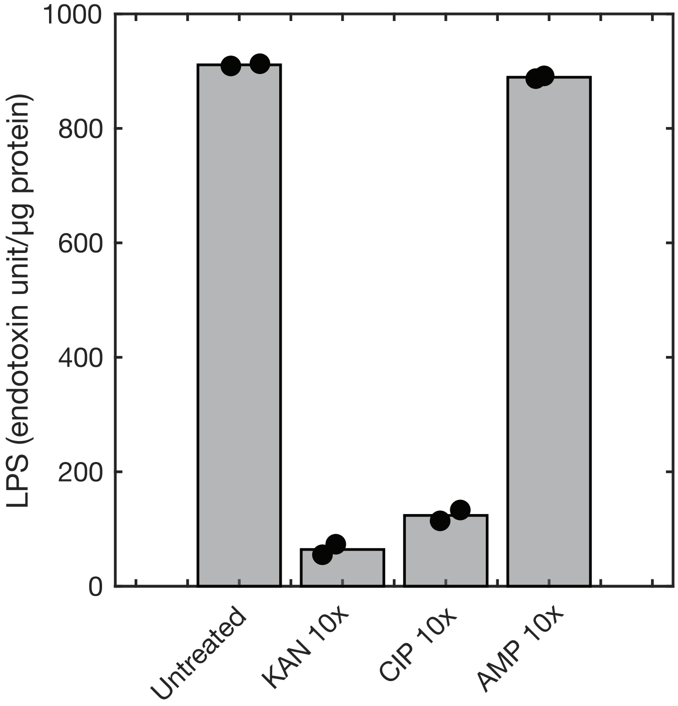

Supplementary Figure 13: **Measurements of lipopolysaccharide levels.** Shown are measurements of lipopolysaccharide (LPS) levels of bulk cultures of cells treated by antibiotics (10x MIC), as measured by an LAL assay. For comparison, data from treatment with 10x MIC ampicillin are included. Data from two biological replicates (black points), and bars indicate averages.

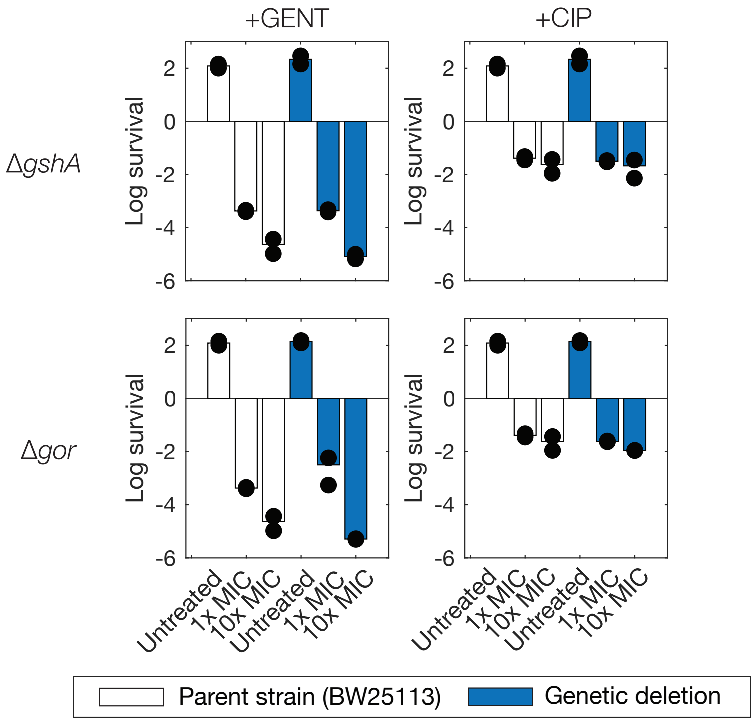

Supplementary Figure 14: **Effects of genetic deletions on antibiotic killing.** Shown are log survival values of *E. coli* under gentamicin and ciprofloxacin treatment, as determined by CFU plating and counting after 4 h of treatment. Δ*gshA* and Δ*gor* strains from the Keio collection, as well as the parent strain (BW25113), were used. Data from two biological replicates (black points), and bars indicate averages. Positive values indicate increases in CFU/mL.

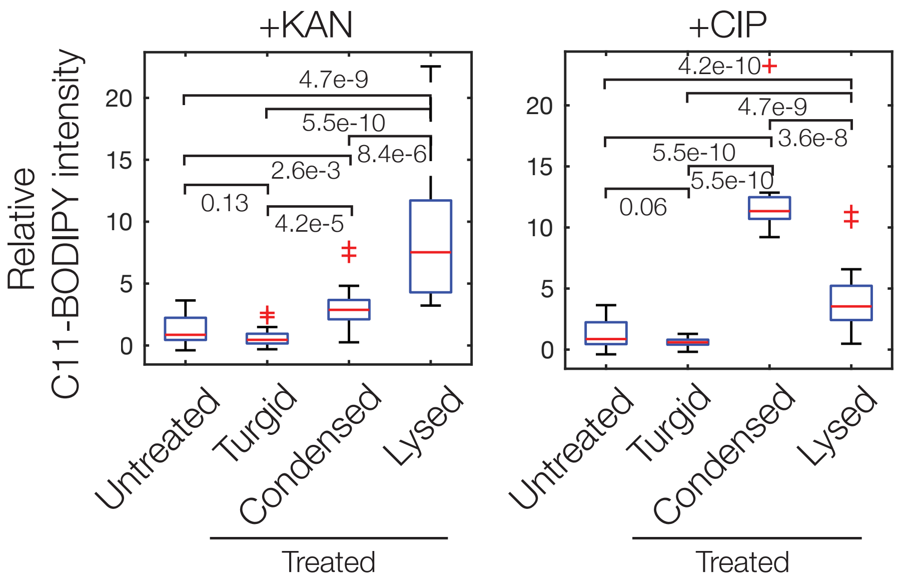

Supplementary Figure 15: **Measurements of C11-BODIPY fluorescence in the presence of α-tocopherol.** Shown are fluorescence intensities of antibiotic-treated *E. coli* pretreated by the lipophilic antioxidant α-tocopherol (50 mM), and in the presence of the lipid peroxidation-sensitive dye C11-BODIPY. The kanamycin and ciprofloxacin concentrations used were 10x MIC. Data are from 20 cells in each group. Box plots indicating the median (center), 25th percentile and 75th percentile (bounds of box), and extreme data points not considered outliers (bounds of whiskers) are shown, and red crosses indicate outliers including the minimum and maximum values. p-values for two-sample Kolmogorov-Smirnov tests are shown next to corresponding brackets.

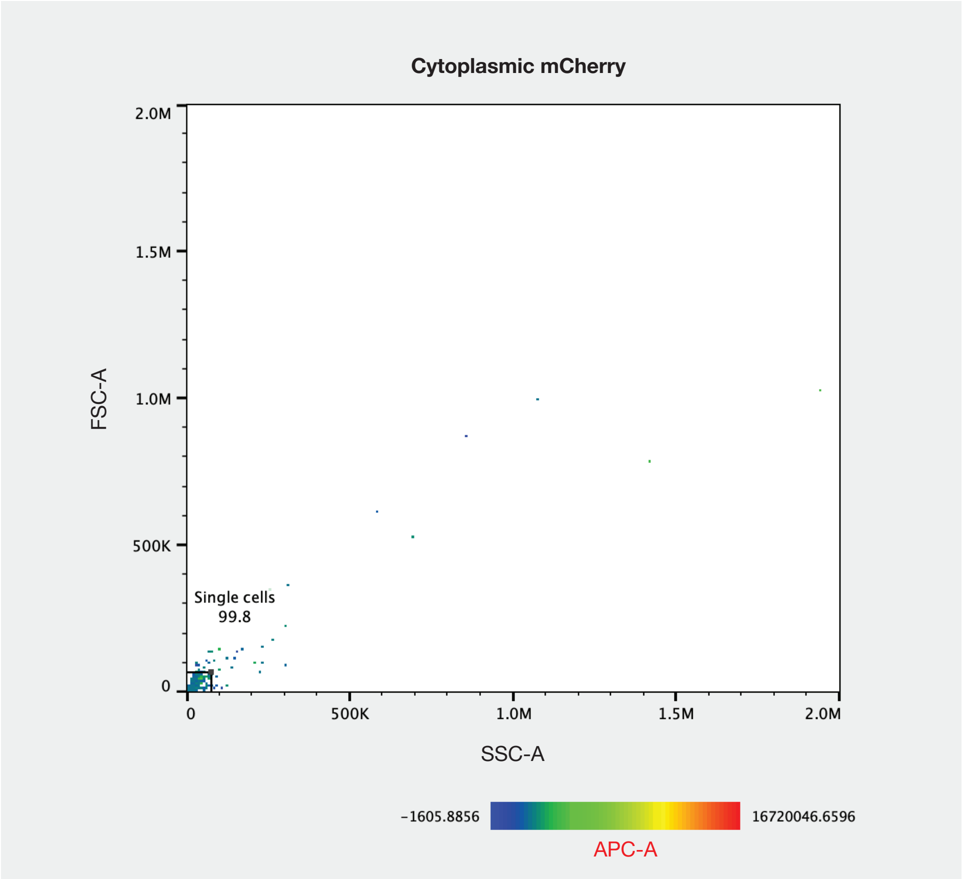

Supplementary Figure 16: **Gating strategy for flow cytometry.** *E. coli* cells containing cytoplasmic mCherry were filtered by calibration of forward- and side-scattering area measurements (FSC-A and SSC-A) using the APC-A (red) fluorochrome. 20,000 scattering events are shown, and results are representative of four biological replicates with 20,000 scattering events each. The gating strategy was used for the flow cytometry measurements shown in Fig. 4 of the main text and Supplementary Figs. 1 and 8.

Supplementary References

1. Wong, F. & Amir, A. Mechanics and dynamics of bacterial cell lysis. *Biophys. J.* **116**, 2378–2389 (2019).
2. Dwyer, D. J. *et al.* Antibiotics induce redox-related physiological alterations as part of their lethality. *Proc. Natl. Acad. Sci. USA* **111**, E2100–E2109 (2014).
3. Yao, Z., Kahne, D. & Kishony, R. Distinct single-cell morphological dynamics under beta-lactam antibiotics. *Mol. Cell* **48**, 705–712 (2012).
4. Whatmore, A. M. & Reed, R. H. Determination of turgor pressure in *Bacillus subtilis*: a possible role for K+ in turgor regulation. *J. Gen. Microbiol.* **136**, 2521–2526 (1990).
5. Sun, Y., Sun, T.-L. & Huang, H. W. Physical properties of *Escherichia coli* spheroplast membranes. *Biophys. J.* **107**, 2082–2090 (2014).
6. Dougherty, T. J. & Saukkonen, J. J. Membrane permeability changes associated with DNA gyrase inhibitors in *Escherichia coli*. *Antimicrob. Agents Chemother.* **28**, 200–206 (1985).
7. Diver, J. M. & Wise, R. Morphological and biochemical changes in *Escherichia coli* after exposure to ciprofloxacin. *J. Antimicrob. Chemother.* **18**, 31–41 (1986).
8. Elliott, T. S., Shelton, A. & Greenwood, D. The response of *Escherichia coli* to ciprofloxacin and norfloxacin. *J. Med. Microbiol.* **23**, 83–88 (1987).
9. Wang, S., Arellano-Santoyo, H., Combs, P. A. & Shaevitz, J. W. Actin-like cytoskeleton filaments contribute to cell mechanics in bacteria. *Proc. Natl. Acad. Sci. USA* **107**, 9182–9185 (2010).
10. Koch, A. L. *Bacterial growth and form* (Springer Science & Business Media, 2001).
11. Hussain, S. *et al.* MreB filaments create rod shape by aligning along principal membrane curvature. *eLife* **7**, e32471 (2018).
12. Wang, S. & Wingreen, N. S. Cell shape can mediate the spatial organization of the bacterial cytoskeleton. *Biophys. J.* 541–552 (2013).
13. *et al.*, K. A. S. Protein diffusion in the periplasm of *e. coli* under osmotic stress. *Biophys J.* 22–31 (2011).
14. Silhavy, T. J., Kahne, D. & Walker, S. The bacterial cell envelope. *Cold Spring Harb. Perspect. Biol.* **2**, a000414 (2010).
15. Movva, N. R., Nakamura, K. & Inouye, M. Regulatory region of the gene for the ompA protein, a major outer membrane protein of *Escherichia coli*. *Proc. Natl. Acad. Sci. USA* **77**, 3845–3849 (1980).
16. Cussler, E. L. *Diffusion: Mass Transfer in Fluid Systems* (Cambridge University Press, 1997).
17. Sperelakis, N. *Cell Physiology Source Book: Essentials of Membrane Biophysics* (Academic Press, 1995).
18. Phillips, R., Kondev, J., Theriot, J. & Garcia, H. *Physical Biology of the Cell* (Taylor and Francis, 2012).
19. Çetiner, U. *et al.* Tension-activated channels in the mechanism of osmotic fitness in *Pseudomonas aeruginosa*. *J. Gen. Physiol.* **149**, 595–609 (2017).
20. Deng, Y., Sun, M. & Shaevitz, J. W. Direct measurement of cell wall stress stiffening and turgor pressure in live bacterial cells. *Phys. Rev. Lett.* **107**, 158101 (2011).
21. Amir, A., Babaeipour, F., McIntosh, D. B., Nelson, D. R. & Jun, S. Bending forces plastically deform growing bacterial cell walls. *Proc. Natl. Acad. Sci. USA* **111**, 5778–5783 (2014).
22. Yao, X., Jericho, M., Pink, D. & Beveridge, T. Thickness and elasticity of Gram-negative murein sacculi measured by atomic force microscopy. *J. Bacteriol.* **181**, 6865–6875 (1999).
23. Cayley, D. S., Guttman, H. J. & Record, M. T. Biophysical characterization of changes in amounts and activity of *Escherichia coli* cell and compartment water and turgor pressure in response to osmotic stress. *Biophys. J.* **78**, 1748–1764 (2000).
