# Supplementary material for: Cytoplasmic condensation induced by membrane damage is associated with antibiotic lethality
Source: Nat Commun. 2021 Apr 19;12:2321. doi: 10.1038/s41467-021-22485-6 (PMC8055701; doi:10.1038/s41467-021-22485-6)
Supplement: Supplementary file 3 — Description of Additional Supplementary Files [file 41467_2021_22485_MOESM3_ESM.pdf]

Description of Additional Supplementary Files

File Name: Supplementary Movie 1
Description: Long-term microfluidic imaging of a population of E. coli cells treated by kanamycin. Supplementary Movie 1 shows a population of E. coli MG1655 cells in the time after kanamycin treatment at 10x MIC. The time is indicated in hours:minutes:seconds, and the timelapse covers a period of about 11.5 hrs. All videos have LB as the growth medium, and long-term imaging was performed inside a microfluidic chip. Antibiotic treatment at 1x and 100x MIC exhibited similar phenotypes; see Supplementary Fig. 4.

File Name: Supplementary Movie 2
Description: Long-term microfluidic imaging of a population of E. coli cells treated by ciprofloxacin. Supplementary Movie 2 shows a population of E. coli MG1655 cells in the time after ciprofloxacin treatment at 10x MIC. The time is indicated in hours:minutes:seconds, and the timelapse covers a period of 18 hrs. Antibiotic treatment between 0.1x and 100x MIC exhibited similar phenotypes; at 100x MIC, however, less filamentation occurs (see Supplementary Fig. 4 and Supplementary Movie 3). Scale bar, 20 µm.

File Name: Supplementary Movie 3
Description: Long-term microfluidic imaging of a population of E. coli cells treated by ciprofloxacin. Supplementary Movie 3 shows a population of E. coli MG1655 cells in the time after ciprofloxacin treatment at 100x MIC. The time is indicated in hours:minutes:seconds, and the timelapse covers a period of about 22 hrs.

File Name: Supplementary Movie 4
Description: E. coli cells after kanamycin treatment and washout. Supplementary Movie 4 shows a population of E. coli MG1655 cells in the time after kanamycin treatment (treatment for ~30 minutes) at 10x MIC and washout. The time is indicated in hours:minutes:seconds, and the timelapse covers a period of about 6.5 hrs.

File Name: Supplementary Movie 5
Description: E. coli cells after ciprofloxacin treatment and washout. Supplementary Movie 5 shows a population of E. coli MG1655 cells in the time after ciprofloxacin treatment (treatment for ~30 minutes) at 10x MIC and washout. The time is indicated in hours:minutes:seconds, and the timelapse covers a period of about 2.5 hrs.

File Name: Supplementary Movie 6
Description: E. coli cells after kanamycin treatment and exogenous supplementation of glutathione. Supplementary Movie 6 shows a population of E. coli MG1655 cells treated with kanamycin at 10x MIC, and pretreated with 10 mM glutathione. The time is indicated in hours:minutes:seconds, and the timelapse covers a period of about 5 hrs. Scale bar, 10 µm.

File Name: Supplementary Movie 7
Description: E. coli cells after ciprofloxacin treatment and exogenous supplementation of glutathione. Supplementary Movie 7 shows a population of E. coli MG1655 cells treated

with ciprofloxacin at 10x MIC, and pretreated with 10 mM glutathione. The time is indicated in hours:minutes:seconds, and the timelapse covers a period of about 9 hrs.

File Name: Supplementary Movie 8
Description: Untreated E. coli cells after application of a 250 mM hypoosmotic shock. Supplementary Movie 8 shows a population of E. coli W3110 cells in the time immediately after application of a 250 mM hypoosmotic downshift from LB+sorbitol to LB only. The time between frames is 0.5 s. Note that the cell volumes recover from hypoosmotic shock around frame 300, consistent with previously quantified cell volume trajectories after hypoosmotic shock (ref. 41 of the main text).

File Name: Supplementary Movie 9
Description: E. coli cells after ciprofloxacin treatment and application of a 250 mM hypoosmotic shock. Supplementary Movie 9 shows a population of E. coli W3110 cells 4 hrs after ciprofloxacin treatment at 10x MIC, in the time immediately after application of a 250 mM hypoosmotic downshift from LB+sorbitol+drug to LB+drug only. The time between frames is 1 s.

File Name: Supplementary Movie 10
Description: E. coli cells after kanamycin treatment and application of a 250 mM hypoosmotic shock. Supplementary Movie 10 shows a population of E. coli W3110 cells 4 hrs after kanamycin treatment at 10x MIC, in the time immediately after application of a 250 mM hypoosmotic downshift from LB+sorbitol+drug to LB+drug only. The time between frames is 1 s.
