# Supplementary material for: Cytoplasmic condensation induced by membrane damage is associated with antibiotic lethality
Source: Nat Commun. 2021 Apr 19;12:2321. doi: 10.1038/s41467-021-22485-6 (PMC8055701; doi:10.1038/s41467-021-22485-6)
Supplement: Supplementary file 14 — Reporting Summary [file 41467_2021_22485_MOESM14_ESM.pdf]

nature research

Corresponding author(s): James J. Collins

Last updated by author(s): Mar 4, 2021

Reporting Summary

Nature Research wishes to improve the reproducibility of the work that we publish. This form provides structure for consistency and transparency in reporting. For further information on Nature Research policies, see our Editorial Policies and the Editorial Policy Checklist.

Statistics

For all statistical analyses, confirm that the following items are present in the figure legend, table legend, main text, or Methods section.

n/a	Confirmed	
☐	☒	The exact sample size (n) for each experimental group/condition, given as a discrete number and unit of measurement
☐	☒	A statement on whether measurements were taken from distinct samples or whether the same sample was measured repeatedly
☐	☒	The statistical test(s) used AND whether they are one- or two-sided<br>*Only common tests should be described solely by name; describe more complex techniques in the Methods section.*
☒	☐	A description of all covariates tested
☒	☐	A description of any assumptions or corrections, such as tests of normality and adjustment for multiple comparisons
☐	☒	A full description of the statistical parameters including central tendency (e.g. means) or other basic estimates (e.g. regression coefficient) AND variation (e.g. standard deviation) or associated estimates of uncertainty (e.g. confidence intervals)
☐	☒	For null hypothesis testing, the test statistic (e.g. F, t, r) with confidence intervals, effect sizes, degrees of freedom and P value noted<br>*Give P values as exact values whenever suitable.*
☒	☐	For Bayesian analysis, information on the choice of priors and Markov chain Monte Carlo settings
☒	☐	For hierarchical and complex designs, identification of the appropriate level for tests and full reporting of outcomes
☒	☐	Estimates of effect sizes (e.g. Cohen's d, Pearson's r), indicating how they were calculated

Our web collection on statistics for biologists contains articles on many of the points above.

Software and code

Policy information about availability of computer code

Data collection	All software used for data collection are described in detail in the Methods section. Briefly, Zen Blue Lite (Zeiss), NIS-Elements (v4.60.00, Nikon), and AxioVision (v4.8, Zeiss) were used for microscopic image acquisition and ImageJ (v2.0, NIH) was used for processing and analysis. Manufacturer software from the CellASIC ONIX platform (MilliporeSigma) was used for microfluidic experiments, and the Nanowizard IV (JPK Instrument) Quantitative Imaging mode was used for atomic force microscopy. CytoFLEX software from Beckman Coulter (CytExpert, v2.4) was used for flow cytometry, and manufacturer software from Molecular Devices for the SpectraMax M3 was used for plate reader measurements.
Data analysis	ImageJ (v2.0, NIH) and Adobe Photoshop (Adobe) were used for image processing and analysis. FlowJo (v10, Becton Dickinson) was used for flow cytometry data processing and analysis. MATLAB (v2019b, Mathworks) was used for data processing of single-cell traces, and to augment the mathematical modeling performed in our work.

For manuscripts utilizing custom algorithms or software that are central to the research but not yet described in published literature, software must be made available to editors and reviewers. We strongly encourage code deposition in a community repository (e.g. GitHub). See the Nature Research guidelines for submitting code & software for further information.

Data

Policy information about availability of data

All manuscripts must include a data availability statement. This statement should provide the following information, where applicable:

- Accession codes, unique identifiers, or web links for publicly available datasets
- A list of figures that have associated raw data
- A description of any restrictions on data availability

Source data are provided with this paper.

Field-specific reporting

Please select the one below that is the best fit for your research. If you are not sure, read the appropriate sections before making your selection.

☒ Life sciences ☐ Behavioural & social sciences ☐ Ecological, evolutionary & environmental sciences

For a reference copy of the document with all sections, see nature.com/documents/nr-reporting-summary-flat.pdf

Life sciences study design

All studies must disclose on these points even when the disclosure is negative.

Sample size	Sample sizes were typically chosen based on the availability of experimental data. For single-cell measurements, all cells for which we could perform measurements were used, with the exception of Fig. 2, for which we analyzed only 30 cells, and Fig. 3, for which we analyzed 10 to 20 cells. We found that these limited sample sizes provided sufficient data for the statistical tests described. For bulk culture experiments, all data from all experiments were used.
Data exclusions	When performing the single-cell measurements of Figs. 2 and 3 described above, we chose the first 20 to 30 timelapses to perform cell length measurements on, because the smaller sample sizes provided sufficient data for the statistical tests described. All other timelapses were excluded in a pre-established manner in these measurements, because they were subsequent timelapses taken from the same treatment group of cells. For all other measurements and experiments, no data were excluded.
Replication	All data were representative of at least two biological replicates, and the numbers of replications of each experiment are indicated as relevant. All attempts at replication were successful.
Randomization	There were no preallocation considerations. We chose cell cultures to be subjected to treatment or control randomly.
Blinding	As we were not aware of any potential sources of bias in our experiments, we were not blinded to allocation when performing experiments or performing measurements.

Reporting for specific materials, systems and methods

We require information from authors about some types of materials, experimental systems and methods used in many studies. Here, indicate whether each material, system or method listed is relevant to your study. If you are not sure if a list item applies to your research, read the appropriate section before selecting a response.

Materials & experimental systems

n/a	Involved in the study	
☒	☐	Antibodies
☒	☐	Eukaryotic cell lines
☒	☐	Palaeontology and archaeology
☒	☐	Animals and other organisms
☒	☐	Human research participants
☒	☐	Clinical data
☒	☐	Dual use research of concern

Methods

n/a	Involved in the study	
☒	☐	ChIP-seq
☐	☒	Flow cytometry
☒	☐	MRI-based neuroimaging

Flow Cytometry

Plots

Confirm that:

☒ The axis labels state the marker and fluorochrome used (e.g. CD4-FITC).

☒ The axis scales are clearly visible. Include numbers along axes only for bottom left plot of group (a 'group' is an analysis of identical markers).

☐ All plots are contour plots with outliers or pseudocolor plots.

☒ A numerical value for number of cells or percentage (with statistics) is provided.

Methodology

Sample preparation	Bacterial cells were grown as described in Methods and pipetted directly into 96-well plates.
Instrument	We used a CytoFLEX S flow cytometer from Beckman Coulter.

Software	We used the manufacturer software from Beckman Coulter (CytExpert, v2.4) for data acquisition. FlowJo (v10, Becton Dickinson) was used for downstream data processing and analysis.
Cell population abundance	As bacterial cells were grown in the absence of any matrix or impurities in LB broth, we found that most scattering events corresponded to bacterial cells. We verified this by calibrating forward and side-scattering events with respect to the standard determined by a fluorescent cytoplasmic mCherry strain, as noted in the Methods section.
Gating strategy	Gating was based on FSC-A and SSC-A (area) measurements, according to the strategy shown in Supplementary Fig. 16. Fluorescence was then measured with respect to the fluorophores FITC (green), Pb450 (blue), and APC-A (red).

☒ Tick this box to confirm that a figure exemplifying the gating strategy is provided in the Supplementary Information.
